# Artificial selection on *GmOLEO1* contributes to the increase in seed oil during soybean domestication

**DOI:** 10.1371/journal.pgen.1008267

**Published:** 2019-07-10

**Authors:** Dan Zhang, Hengyou Zhang, Zhenbin Hu, Shanshan Chu, Kaiye Yu, Lingling Lv, Yuming Yang, Xiangqian Zhang, Xi Chen, Guizhen Kan, Yang Tang, Yong-Qiang Charles An, Deyue Yu

**Affiliations:** 1 Collaborative Innovation Center of Henan Grain Crops, College of Agronomy, Henan Agricultural University, Zhengzhou, China; 2 Department of Biological Sciences, University of North Carolina at Charlotte, Charlotte, North Carolina, United States of America; 3 Department of Agronomy, Kansas State University, Manhattan, Kansas, United States of America; 4 National Center for Soybean Improvement, National Key Laboratory of Crop Genetics and Germplasm Enhancement, Jiangsu Collaborative Innovation Center for Modern Crop Production, Nanjing Agricultural University, Nanjing, China; 5 School of Life Sciences, Guangzhou University, Guangzhou, China; 6 USDA-ARS, Plant Genetics Research Unit at Donald Danforth Plant Science Center, St. Louis, Missouri, United States of America; The University of North Carolina at Chapel Hill, UNITED STATES

## Abstract

Increasing seed oil content is one of the most important breeding goals for soybean due to a high global demand for edible vegetable oil. However, genetic improvement of seed oil content has been difficult in soybean because of the complexity of oil metabolism. Determining the major variants and molecular mechanisms conferring oil accumulation is critical for substantial oil enhancement in soybean and other oilseed crops. In this study, we evaluated the seed oil contents of 219 diverse soybean accessions across six different environments and dissected the underlying mechanism using a high-resolution genome-wide association study (GWAS). An environmentally stable quantitative trait locus (QTL), *GqOil20*, significantly associated with oil content was identified, accounting for 23.70% of the total phenotypic variance of seed oil across multiple environments. Haplotype and expression analyses indicate that an oleosin protein-encoding gene (*GmOLEO1*), colocated with a leading single nucleotide polymorphism (SNP) from the GWAS, was significantly correlated with seed oil content. *GmOLEO1* is predominantly expressed during seed maturation, and GmOLEO1 is localized to accumulated oil bodies (OBs) in maturing seeds. Overexpression of *GmOLEO1* significantly enriched smaller OBs and increased seed oil content by 10.6% compared with those of control seeds. A time-course transcriptomics analysis between transgenic and control soybeans indicated that *GmOLEO1* positively enhanced oil accumulation by affecting triacylglycerol metabolism. Our results also showed that strong artificial selection had occurred in the promoter region of *GmOLEO1*, which resulted in its high expression in cultivated soybean relative to wild soybean, leading to increased seed oil accumulation. The *GmOLEO1* locus may serve as a direct target for both genetic engineering and selection for soybean oil improvement.

## Author summary

Soybean seed oil is an important quality trait targeted during domestication and breeding. However, the molecular mechanism of soybean oil regulation is largely unknown due to its complex genetic architecture and environmental sensitivity. In this paper, we integrated GWAS across multiple environments, haplotype analysis, genetic transformation, and diversity analysis to study the genetic architecture of oil content and the underlying mechanism in soybean. This combined analysis enabled us to identify an environmentally stable QTL (*GqOil20*) and functionally verified that *GmOLEO1* positively regulates total seed oil accumulation in soybean seeds. In addition, we found that *GmOLEO1* showed a higher level of expression in cultivated soybean seeds than in wild soybean seeds, possibly as the result of the positive selection of the promoter, resulting in seed oil accumulation. Moreover, we identified an elite *GmOLEO1* haplotype that correlated strongly with high oil content in soybean, holding great potential for assisting oil improvement in soybean breeding. Our study provided a new genetic resource for oil content improvement in soybean and other oilseed crops.

## Introduction

Soybean (*Glycine max* (L.) Merr.) is an important food and oil crop. Soybean seeds accumulate large amounts of oil and protein and have been intensively targeted for human consumption during long-term domestication and cultivation. Given the high percentage of oil in soybean seeds, the demand for soybean oil production has increased dramatically due to the increasing demand for vegetable oils and expanded use of biodiesel, and the seed composition improvement is of particular interest in terms of increasing awareness of health issues around dietary fats [1]. However, oil accumulation in the seed is a complex metabolic process that is environmentally sensitive; thus, stably expressed oil-enhancing key genes that can be applied to soybean molecular breeding have rarely been reported, and the mechanism of the variance of oil content in soybean remains largely unknown.

In plants, accumulated oil in seeds is generally stored as triacylglycerols (TAGs). TAG synthesis is initiated from glucose in the cytosol, and the resulting products from glycolysis are transported into the plastid for fatty acid (FA) synthesis. The FAs are processed by a series of key enzymes to produce C_16:0_ and C_18:0_ acyl chains and desaturated products, such as C18:1. FA products are then exported to the endoplasmic reticulum (ER) to form TAGs via the acyl-CoA-dependent and acyl-CoA-independent pathways [2]. The resulting TAGs are present in subcellular spherical lipid droplets in various plant tissues; the lipid droplets stored in seeds are usually called oil bodies (OBs) and have been extensively investigated previously in studies of, for instance, the structure and composition of an OB and the essential role of OB-related proteins, such as oleosins, in OB formation, mobilization, and oil accumulation [3–10]. Previous studies have indicated that oleosins play conserved roles in OB formation in seeds in several oilseed plants [7–8]. Suppression of a soybean oleosin produces micro-OBs [10], while the effects of OBs on seed oil accumulation have rarely been reported in soybean. The soybean genome contains 13 putative oleosin-encoding genes [11], and if any of them are involved in oil accumulation remain unexploited.

By linkage and linkage disequilibrium mapping, over 300 quantitative trait loci (QTLs) associated with seed oil content have been identified across all 20 chromosomes in the soybean genome over the past decades (SoyBase, https://soybase.org). These studies have revealed the polygenic nature of oil regulation, and the majority of loci were found to have varying additive, epistatic or QTL×environment effects [12–15], implying that traditional breeding based on genetic crossing and phenotypic selection may be inadequate for oil improvement. Recent studies have shown that increased oil in soybean could be achieved by genetic engineering of transcription factors involved in oil accumulation [16–18] or a QTL gene controlling seed coat bloom [19]. However, QTLs directly related to seed oil accumulation in soybean have not been cloned; thus, the underlying mechanism has not been thoroughly elucidated to date. Therefore, identifying an environmentally stable major QTL regulating seed oil content is urgently needed to substantially enhance seed oil content and understand the underlying regulatory mechanism in soybean.

To reveal the genetic basis of seed oil content and elucidate how oil accumulation is regulated, we investigated the oil content variation in 219 diverse soybean genotypes across six different environments and conducted a high-density genome-wide association study (GWAS) using 201,994 genome-wide single nucleotide polymorphisms (SNP). In total, three QTLs were identified to be significantly associated with soybean oil content across at least two environments, with *GqOil20* on chromosome 20 stably expressing across all six environments. We also found that an oleosin-encoding gene, *GmOLEO1*, in the *GqOil20* linkage disequilibrium (LD) block was exclusively expressed in developing seeds and that its expression level was significantly correlated with oil content within selected genotypes. We subsequently verified that *GmOLEO1* contributed to oil accumulation in soybean seeds by conducting a series of molecular assays. Our results reveal an environmentally stable QTL/gene controlling oil accumulation in soybean seeds, provide new insight into oil accumulation in soybean and offer new directions for breeding soybean varieties with enhanced seed oil content.

## Results

### GWAS identified a stably expressed QTL associated with oil content

To identify the genetic variation in seed oil content, we measured the oil contents of 219 soybean genotypes with diverse genetic backgrounds across six different environments. Seed oil content exhibited large amounts of natural variation within the association panel in each environment and showed relative consistency across the six environments (S1A and S1B Fig, S1 Table). The mean oil content for the 219 accessions ranged from 18.10% to 18.97% across the six environments, and the observed maximum oil content reached 27.69% in Environment 1 (E1), which was approximately three times higher than the minimum value (9.64%) observed in E6 (S1A Fig, S1 Table). The distribution of oil content for the association panel in each environment was approximately normal (S1C Fig). Analysis of variance (ANOVA) indicated a significant difference (*p* < 0.001) in oil content among the genotypes, the oil content was significantly affected by environments (*p* < 0.001) (S1 Table), and the heritability was 0.64.

**Fig 1:**
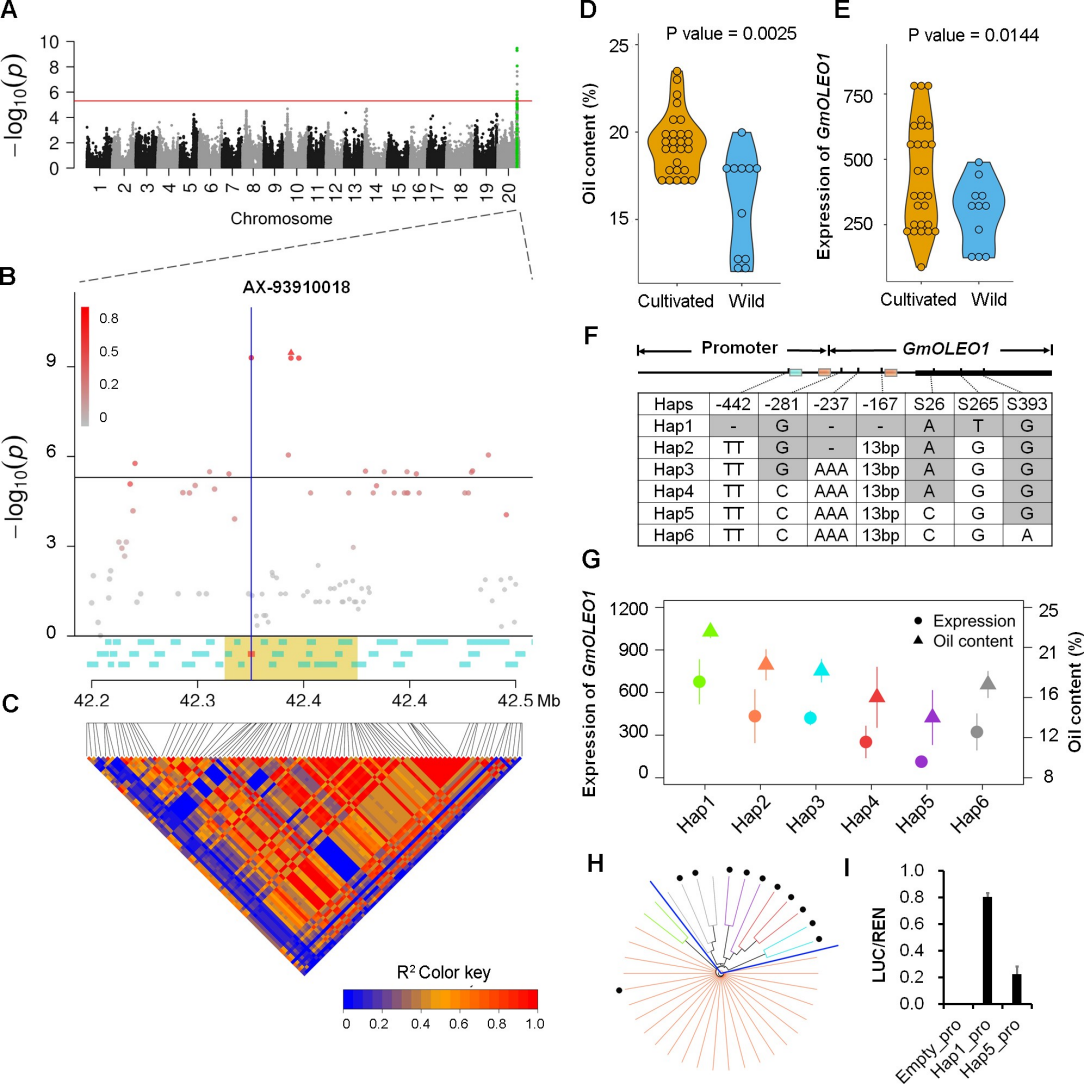
GWAS for oil content in soybean seeds and candidate gene selection analyses. A, A Manhattan plot for the BLUP of soybean oil content across six environments by association mapping. A red horizontal line depicts the Bonferroni-adjusted significance threshold (*P*<4.95×10^−6^). The x-axis shows the 20 soybean chromosomes, and the y-axis shows the significance expressed as the -log_10_*P* value. B, A zoomed-in Manhattan plot of the 0.2-Mb genomic region on either side of the most significant SNP at the QTL *GqOil20* on chromosome 20. The red solid triangle represents the leading SNP (AX-93910018). The color intensity of other SNPs is shown according to their LDs (*r*^2^ value) with the leading SNP. Gene models within the region are indicated with blue rectangles, and the red rectangle represents the candidate gene *Glyma*.*20G196600*. The 50-kb genomic regions on both sides of the leading SNP are highlighted in light yellow. C, The extent of linkage disequilibrium (LD) in the 0.2-Mb genomic region on either side of the leading SNP based on pairwise *r*^*2*^ values. The *r*^*2*^ values are indicated using the color intensity index. D, Comparisons of seed oil content (%) between cultivated and wild soybeans. E, Comparison of *GmOLEO1* expression between cultivated and wild soybeans. F, haplotypes of *GmOLEO1* among 38 soybean genotypes. The orange and cyan rectangles on the promoter region indicate the cis-acting regulatory elements involved in ABA response and seed-specific regulation, respectively. G, Comparative analyses of the *GmOLEO1* expression and oil content between the six different haplotypes. H, A neighbor-joining tree of the 38 accessions using variants from *GmOLEO1*. The edge color pattern was the same as that indicated by the six different haplotypes in (**G**). Black solid dots represent *G*. *soja* accessions. I, Ratios of LUC and REN activity in Arabidopsis protoplasts transformed with recombinant plasmids containing the *GmOLEO1* promoters from two different haplotypes (Hap1_pro, Hap5_pro) and the control vector. Significance analysis was performed using Fisher’s protected least significant difference (LSD) test. ** indicates a significant difference at the 0.01 level.

Because of the wide variation in seed oil content in the panel across the environments, we performed GWAS for the oil content in six environments (E1 to E6) and the best linear unbiased prediction (BLUP) using 201,994 genome-wide SNPs with a minor allele frequency (MAF) ≥ 0.05 in an effort to identify the genetic loci associated with soybean oil content. In total, 110 SNPs on three chromosomes (8, 12, and 20) were identified as significantly associated with oil content across at least two environments (S2 Fig, S2 Table). For the sake of simplicity, we empirically classified closely adjacent SNPs located within 5 Mb into one locus, as previously described [20]. The 110 SNPs were classified into three genomic loci, which were subsequently designated *GqOil8*, *GqOil12*, and *GqOil20* (S2 Fig, S2 Table). Of these QTLs, the most significantly associated SNPs were identified in *GqOil20*, which was in physical proximity to oil-related QTLs identified in previous studies (S2 Table) [21–24]. Importantly, *GqOil20* was consistently identified across all the environments and BLUP except E4 (Fig 1A), and it explained 13.4–24.4% of oil variation, representing the most stably expressed QTL for oil content in soybean.

### *GmOLEO1* is a candidate gene for *GqOil20* and encodes oleosin

It is known that oil content is a key domestication trait undergoing artificial selection [25], and the regulatory genes involved were likely selected during domestication. Thus, a comparison of the genetic diversity at the three loci between cultivated soybean (*G*. *max*) and wild soybean (*G*. *soja*), the progenitor of *G*. *max*, could be helpful in determining the most likely regions containing the oil-controlling gene(s). To this end, we calculated genetic differentiation (*Fst*) within the 140 kb regions upstream and downstream of each leading SNP per locus within a group containing this association panel (272 *G*. *max* accessions) and a panel of 122 *G*. *soja* accessions genotyped with the same microarray, as previously described [26]. After the comparison, we found that *Fst* showed variation, and the *Fst* across the entire group (*G*. *max* and *G*. *soja*) was lower than the average *Fst* in the association panel (*G*. *max* only) in two QTLs (*GqOil8* and *GqOil12*). In contrast, most of the *Fst* values for *GqOil20* were significantly higher in the *G*. *max-G*. *soja* group than in the association panel, suggesting that artificial selection might have occurred in this genomic region in relation to oil accumulation (S3 Fig), consistent with the fact that soybean oil content is a domestication trait [25]. In this regard, *GqOil20* likely harbors a gene or genes that have important functions in the regulation of soybean oil accumulation. Thus, we next focused on *GqOil20* to identify oil-related genes.

To identify the candidate gene, we analyzed the LD region harboring the leading SNPs using BLUP as a phenotype. *GqOil20* contained a total of 33 significant SNPs located within a strong LD with an average *r*^2^ = 0.66 (Fig 1C). Of these genes within the LD according to the *G*. *max* Wm82.a2v1 reference genome (https://phytozome.jgi.doe.gov) (S2 Table), we found that a gene, *Glyma*.*20G196600*, encoding a putative oleosin protein, colocated with the significant SNP AX-93661332 (*P* = 4.98 × 10^−10^) (Fig 1A and 1B, S3 Table). *Glyma*.*20G196600* is an ortholog of *Arabidopsis AtOLE1* (AT4G25140), an oleosin-encoding gene with demonstrated roles in oil body formation [9], while other gene models in this block are annotated to be involved in defense responses (S3 Table). Thus, *Glyma*.*20G196600* might be the candidate gene underlying *GqOil20*, and we designated it *GmOLEO1* for further study.

### Genetic variation and expression of *GmOLEO1* correlated with seed oil content

To investigate whether *GmOLEO1* underlies the domestication region *GqOil20*, we examined the expression patterns and sequence variations of *GmOLEO1* alleles in 38 soybean accessions comprising 27 cultivated and 11 wild genotypes with significant differences in oil content between two subgroups (Fig 1D). Consistent with the observed high oil content in *G*. *max* relative to *G*. *soja*, *GmOLEO1* showed significantly higher expression in cultivated soybeans than in wild soybeans (Fig 1D and 1E), indicating a correlation between the transcript abundance of *GmOLEO1* and oil content.

Next, a 2.3-kb genomic region extending from -1,500 bp upstream of the start codon (ATG) to the 3’-untranslated region (UTR) of *GmOLEO1* was sequenced and analyzed. Sequence analyses identified 12 nucleotide variants that divided the 38 germplasm into six haplotypes (Hap), which were clearly classified into two subgroups (cultivated and wild) by a phylogenetic tree (Fig 1H). Moreover, the six haplotypes represented six levels of seed oil content (Fig 1F and 1G), with Hap1 seeds containing the highest oil content. Of the 12 nucleotide variants, seven variants were found to be significantly associated with soybean oil content (Fig 1F), with four located at -442 (A/C/-, *P* = 5.22×10^−4^), -281 (G/C, *P* = 2.83×10^−4^), -237 (AAA/—, *P* = 1.26×10^−3^), and -167 (13-bp insertion/deletion, *P* = 3.13×10^−3^) being detected in the promoter region and three (*P*_*S26*_ = 5.36×10^−3^, *P*_*S265*_ = 0.02, *P*_*S393*_ = 0.04) occurring in the exon. Of these variants, those in the promoter region represent the most significant variation associated with the variation in seed oil, suggesting that resulting differences in the expression of *GmOLEO1* among the haplotypes might account for the oil content variation.

To further determine whether variation in the promoter affected gene expression, we compared the transcriptional activity of the promoters of Hap1 and Hap5 (Hap1_pro and Hap5_pro) using a dual luciferase reporter gene assay. As shown in Fig 1I, Hap1_pro exhibited 3.58-fold higher activity than Hap5_pro, consistent with the observed higher expression of Hap1 than Hap5 (Fig 1I). These results suggest that *GmOLEO1* is a strong candidate for *GqOil20* and that expression level instead of exon variation is an important factor affecting seed oil content.

### GmOLEO1 is localized to OBs and exclusively expressed in maturing seeds

**Fig 2:**
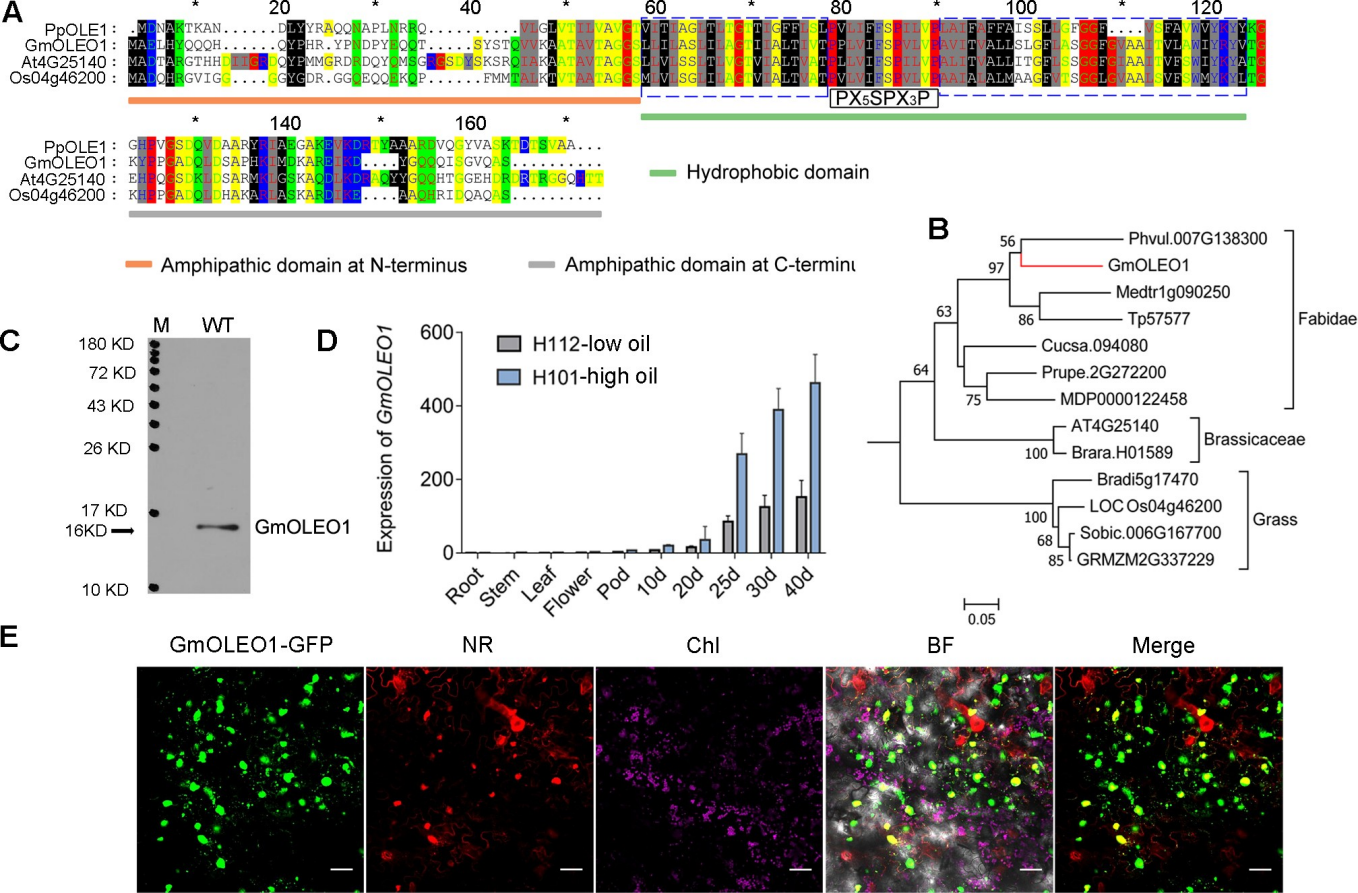
Sequence and expression analysis of *GmOLEO1*. A, Domain structure and full-length amino acid alignment of representative OLE protein orthologs, including the structurally well-studied PpOLE1 (*Physcomitrella patens*), GmOLEO1, At4G25140 (*Arabidopsis thaliana*), and Os04g46200 (*Oryza sativa*). The amphipathic domains at the N-terminus and C-terminus are highlighted by blue and gray solid boxes, respectively. The conserved hydrophobic domain between two amphipathic proteins is shown by a red solid box. Dotted boxes indicate the two hydrophobic arms that can form a hairpin (blue in D); (PX_5_SPX_3_P) indicate the conserved sequence that forms a loop (black in D). B, Phylogenetic relationship between GmOLEO1 and its orthologs. All amino acid sequences were retrieved from Phytozome (https://phytozome.jgi.doe.gov). C, Gel image showing the molecular mass of GmOLEO1 (15.76 KD) using Western blot. M, protein marker, WT, proteins extracted from developing seeds of Williams 82 at 40 DAF. D, The relative transcript level of *GmOLEO1* in different soybean tissues and developing seeds at 10, 20, 25, 30, and 40 days after flowering between the high-oil soybean cultivar H101 and low-oil cultivar H112; ** *P*<0.01 *t*-test. E, Subcellular localization analysis of *GmOLEO1*. *GmOLEO1*-GFP signal (GFP) colocalizes with oil bodies stained with Nile Red (NR). Chl represents chloroplast, BF represents bright field, and Merge shows both GFP and NR. Scale bar, 20 μm. White arrows indicate oil bodies.

Given that *GmOLEO1* was a strong candidate associated with seed oil content, we characterized its protein structure, phylogeny, and expression pattern. BLASTp showed that GmOLEO1 is an ortholog of *Arabidopsis* AtOLE1 (AT4G25140), an oleosin-like protein with demonstrated roles in OB formation [9] (Fig 2A and 2B). Similar to previously described OLE orthologs in other species, GmOLEO1 contains three conserved structural domains (Fig 2A) [5]. Two amphipathic domains are located at the N- and C-termini, respectively, and a hydrophobic domain is located at the center. In the central hydrophobic domain, GmOLEO1 also contains the conserved “proline knot” sequence (PX_5_SPX_3_P), which can form a loop including a hydrophobic hairpin that penetrates into the TAG matrix and two arms located on both sides of the knot (Fig 2A) [5]. This domain organization allows oleosins to be anchored on the surface of an OB, as illustrated in a previous study [3]. Phylogenetic analysis revealed that OLEO-like proteins from the Faboideae, Brassicaceae, and grass clades clustered separately, suggesting functional conservation within the clade and possible functional diversity between clades (Fig 2B). In addition, sodium dodecyl sulfate-polyacrylamide gel electrophoresis (SDS-PAGE) analyses showed that GmOLEO1 has a low molecular mass (~16 KD) (Fig 2C), consistent with previous findings [9, 27]. These results indicated that the uncharacterized gene *GmOLEO1* encodes a putative OB protein that might play roles associated with OB formation or oil accumulation in soybean.

To determine the temporal and spatial expression pattern of *GmOLEO1*, the expression levels of *GmOLEO1* were examined in ten different tissues and two soybean varieties with different seed oil contents (H101, a high-oil variety; H112, a low-oil variety) (Fig 2D). Quantitative real-time PCR (*q*PCR) results showed that *GmOLEO1* transcripts were undetectable in nonseed tissues, including the roots, stems, leaves, and flowers of both varieties, but its transcripts could be detected in developing seeds beginning at the seed-filling stage (Fig 2D). The abundance of *GmOLEO1* transcripts in seeds increased with the number of days after flowering (DAF), with the highest expression level observed in developing seeds at 40 DAF, which was immediately before that seeds had completely matured (Fig 2D). Overall, the expression level of *GmOLEO1* in the developing seeds of H101 was significantly greater than that in H112 seeds at all tested stages. These results indicated that *GmOLEO1* functions specifically during seed maturation and that transcript abundance positively correlated with oil content (Fig 1G).

We next investigated whether GmOLEO1 was spatially related to OBs. We expressed a *35S*::*GmOLEO1*-*GFP* (green fluorescent protein) construct in tobacco (*Nicotiana benthamiana*) leaf epidermal cells by *agro*-infiltration followed by staining with Nile Red, a lipophilic dye used to visualize OBs [4]. Confocal microscopy analysis revealed that GmOLEO1-linked GFP fluorescence and Nile Red fluorescence signal from OBs were colocalized in seed cells (Fig 2E), indicating that GmOLEO1 was localized to accumulated OBs.

Taken together, the results of haplotype analysis, diversity analysis, phylogenetic analysis, expression analysis, and subcellular localization supported the GWAS results and collectively indicated that *GmOLEO1* was a strong candidate gene underlying *GqOil20* associated with oil accumulation in soybean seeds.

### Overexpression of *GmOLEO1* increased seed oil content with pleiotropic effects on seed-related traits

**Fig 3:**
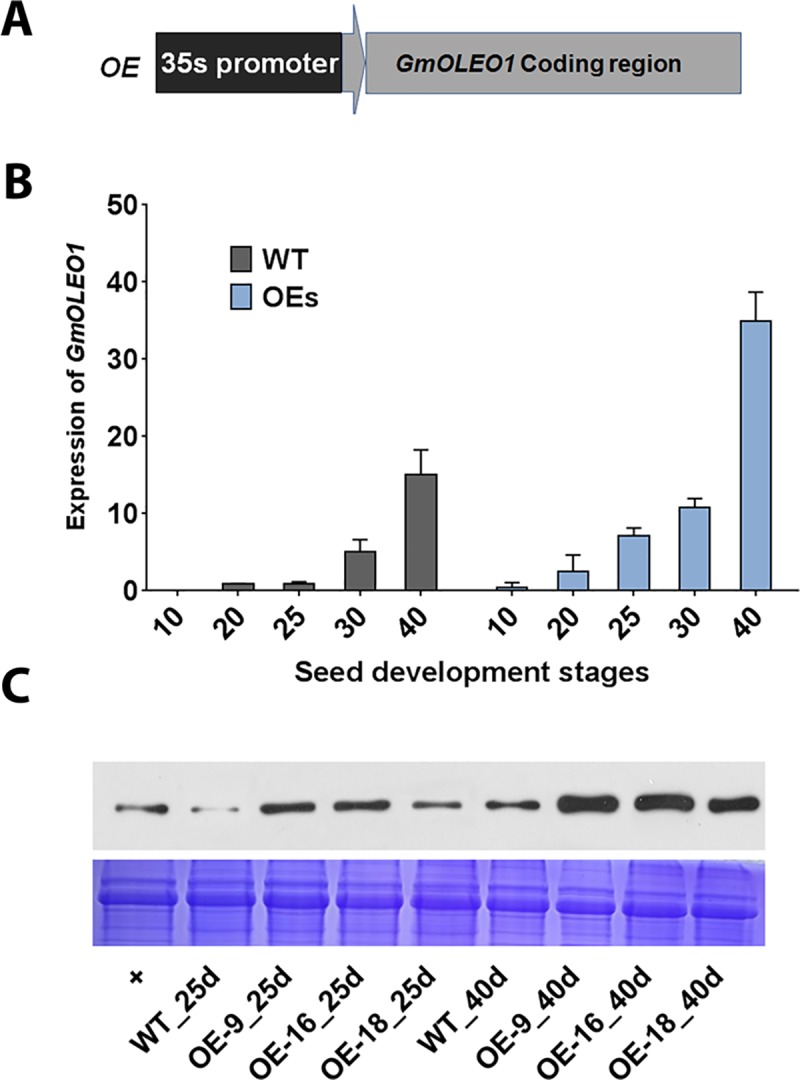
Expression analyses of *GmOLEO1* in transgenic plants. A, Diagram of the plasmid construct (35S::*GmOLEO1*) that was used for soybean transformation. B, Expression analysis of *GmOLEO1* in developing seeds (10, 20, 25, 30, 40 DAF) of T_3_ homozygous OE lines and the wild type (WT). C, Western blot image showing the expression pattern of GmOLEO1 protein between WT and OE seeds at 25 and 40 DAF. Protein bands were detected by Coomassie blue staining (CBB, bottom) or Western blot (WB, top) probed with antibodies to GmOLEO1.

To further demonstrate whether *GmOLEO1* is functionally involved in oil accumulation in soybean seeds, we overexpressed *GmOLEO1* in soybean using an improved cot-node transformation protocol [28]. Successful transformation was determined by detecting both the expression of the selective *bar* gene using the strip test and the presence of 35S::*GmOLEO1* (Fig 3A) using polymerase chain reaction (PCR) analysis in T_0_ plant leaves (S4 Fig). Transgenic soybean lines were self-pollinated through three generations to obtain homozygous lines harboring 35S::*GmOLEO1*. Three independent homozygous transgenic lines (OE-9, OE-16, and OE-18) were selected and used for further analysis.

We first quantified the expression of *GmOLEO1* in developing seeds (10, 20, 25, 30, and 40 DAF). As shown in Fig 3B, *GmOLEO1* expression increased during seed development in both the OE lines and wild type (WT), while its expression in the OE seeds was significantly higher in OE than in WT at each stage of seed development. *GmOLEO1* exhibited a sharp increase in its expression in the OE lines at 25 DAF, which was five days earlier than the increase in expression observed (30 DAF) in WT seeds. In agreement with the observed expression difference between the two soybean varieties described above (Fig 2D), expression of *GmOLEO1* in both the OE lines and WT increased continuously as the seeds developed and reached its highest levels at 40 DAF. This gene expression result was further verified by comparative Western blot analysis of GmOLEO1 protein between WT and the OE lines using an antibody against GmOLEO1. A higher expression of GmOLEO1 was observed in the three OE lines than in the WT at 25 and 40 DAF (Fig 3C).

**Fig 4:**
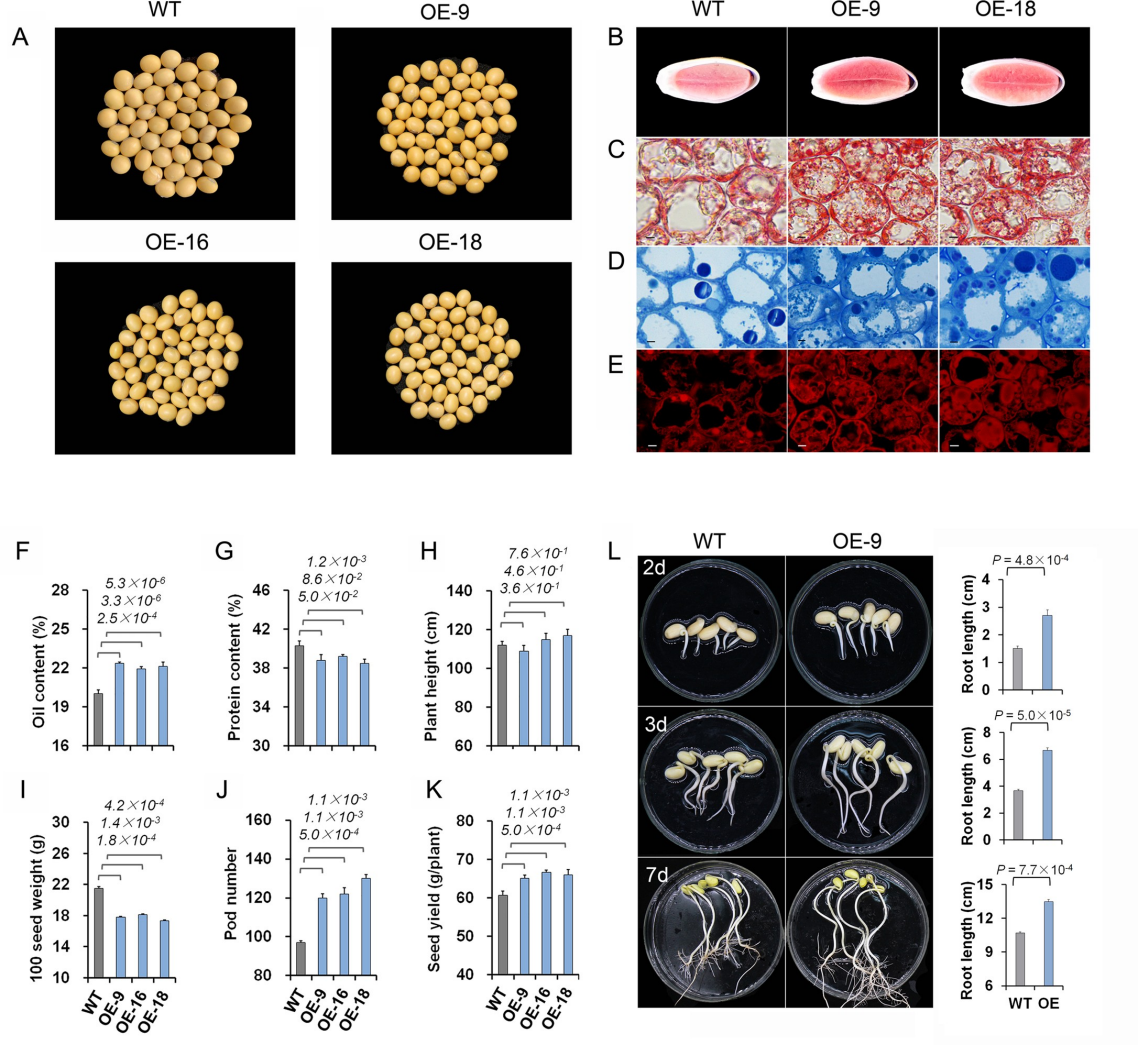
Characterization of transgenic soybeans overexpressing *GmOLEO1*. A, Seed appearance comparison between WT and OE-9, OE-16, OE-18. T_3_ seeds showed a shinier seed surface and smaller seed size than WT. B-E, Microscope-based visualization of lipid and OB accumulation in soybean seeds. B, Cross sections of soybean seeds at 25 DAF showing lipid accumulation as stained with Oil Red O. Two transgenic soybean seeds, OE-9 and OE-18, showed a higher level of TAG accumulation than WT seeds. C and D, Comparison of the abundance of oil bodies (OBs) stained with Oil Red O (C) and alkaline toluidine blue O (D) between the OE lines and WT as visualized using an optical microscope. E, Comparison of the abundance of OBs stained with Nile Red between the OE lines and WT as visualized with a confocal microscope, Bar = 10 μm. F-K, Comparison of seed traits (F and G) and yield-related phenotypes between the OE and WT seeds, including plant height (H), 100-seed weight (I), pod number (J) and seed yield (K) per plant. L, Comparison of seed germination rate and root length between WT and OE-9 at 2, 3 and 7 days postgermination. OE-9 seeds showed faster seed germination than WT seeds. Error bars indicate SD (n = 5). Statistical significance was determined by ANOVA.

Compared with WT, mature seeds from OE lines had shinier surfaces with more yellowish colors and smaller sizes (Fig 4A). The oil contents of the seeds of the three OE lines were 22.35%, 21.91%, and 22.14%, respectively, which were all significantly higher (an absolute average increase of 2.12%, a relative increase of 10.6%, *P* = 4.6 × 10^−6^) than that in WT seeds (20.01%) (Fig 4F). Not surprisingly, the increase in oil content in the OE lines resulted in a significant decrease (*P* = 0.006) in protein content (Fig 4G). To further verify the oil increase in the OE seeds, we conducted a series of microscopy analyses of developing OE seeds (OE-9 and OE-18) at 25 DAF, where sharp increases in the expression of *GmOLEO1* and oleosin were observed (Fig 4C–4F). Microscopy analyses of cross-sections from developing seeds stained with Oil Red O showed that OE seeds have markedly stronger Oil Red O staining than WT seeds, indicating that OE seeds contain a higher level of neutral lipid accumulation than WT seeds (Fig 4B). A further examination of the seed cells using an optical microscope (Nikon, Eclipse Ci, Japan) showed that more OBs were deposited in the two OE lines (OE-9 and OE-18) than in WT (Fig 4C), and a consistent result was found via staining with toluidine blue O (Fig 4D). These observations were further verified by a comparative analysis of Nile Red staining of accumulated oil between OE and WT seeds using a confocal microscope (Nikon, C2, Japan) (Fig 4E). These results visibly illustrated that OE plants overexpressing *GmOLEO1* contained higher levels of oil accumulation in seed cells than WT.

In addition, we observed that overexpression of *GmOLEO1* has pleiotropic effects on other agronomic traits. The phenotypic evaluation indicated that the overexpression resulted in a significant decrease in 100-seed weight in the OE lines compared with that in WT (Fig 4I). However, a significant increase (*P* < 0.01) in pod number per plant and a slight increase in plant height in the OE lines compared with WT lines (Fig 4H and 4J) were observed, which led to an increase (*P* = 0.017) in seed yield per OE plant compared with WT plants (Fig 4K, S4 Table). We also compared the seed germination between two lines. We found that seed germination and root growth were faster in the OE lines than in WT (Fig 4L). These results indicate that *GmOLEO1* is involved in oil accumulation in soybean seeds with pleiotropic effects on yield-related traits, and no yield penalty was found in the current preliminary study.

**Fig 5:**
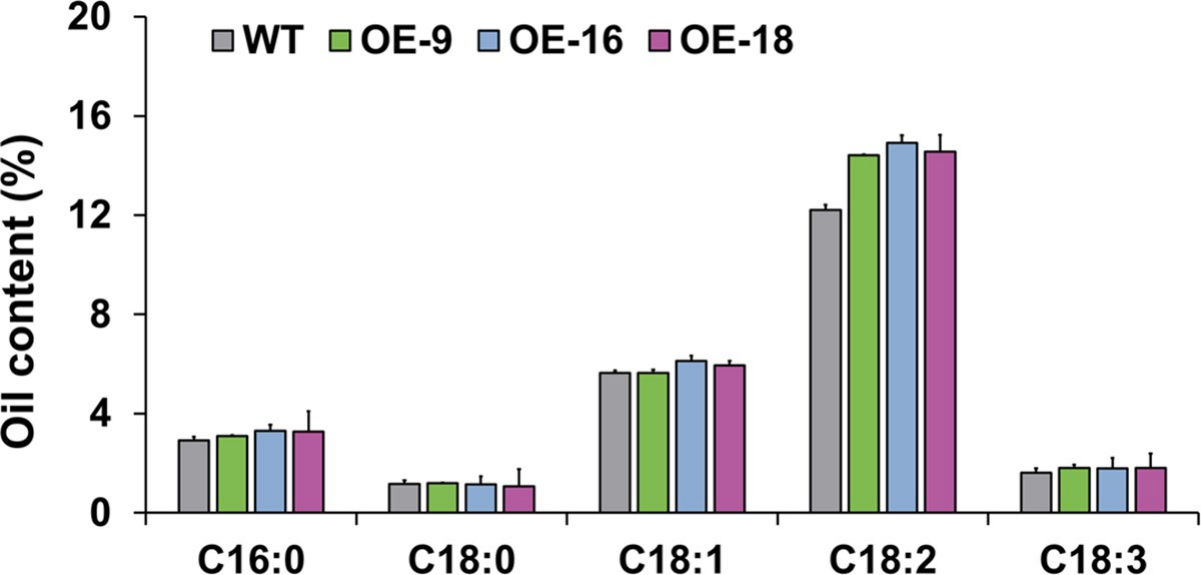
Comparison of fatty acid composition between OE and WT seeds.

In light of the role of *GmOLEO1* in oil accumulation, we further measured and compared the fatty acids between WT and OE seeds to test whether *GmOLEO1* affected FA composition (Fig 5). Compared with the WT, OE seeds contained a higher average total FA content of 12.7% (*P* = 3.2× 10^−4^). Further analysis of five important oil components (TAGs) indicated that two polyunsaturated oil components, linoleic acid (18:2) and linolenic acid (18:3), were significantly increased by 14.4% and 14.9% (*P* = 1.2× 10^−4^ and 9.7× 10^−5^, n = 3), respectively, in the OE seeds compared with WT, while no significant changes in the contents of palmitic acid (16:0), stearic acid (18:0) and oleic acid (18:1) were observed between the OE seeds and WT (Fig 5, S5 Table). This result indicates that the overexpression of *GmOLEO1* also led to increased accumulation of polyunsaturated FAs.

**Fig 6:**
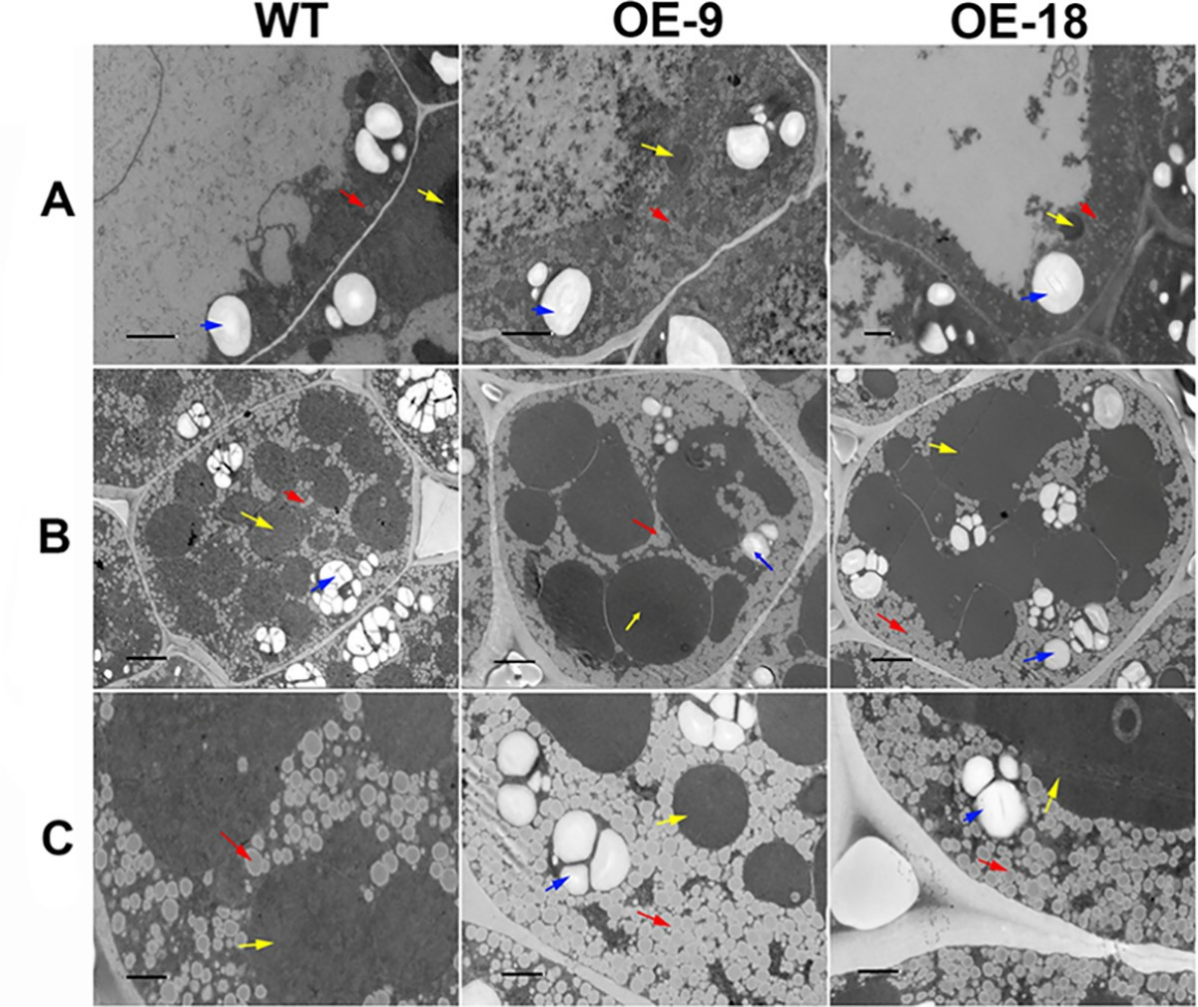
Microscope analyses of OBs in WT and OE seeds. A, OBs in WT and OE seeds at 25 DAF, scale bars = 3 μm. B, Distribution of OBs in a whole seed cell at 40 DAF, scale bars = 5 μm. C, Increased magnification of OB distribution in a seed cell at 40 DAF, scale bars = 2 μm. The seed cells in OE-9 and OE-18 appear to contain smaller OBs than those in WT. Red arrows, oil body; yellow arrows, protein body; blue arrows, starch granules.

Last, we compared the OBs of OE and WT seeds using transmission electron microscopy. At 25 and 40 DAF, the OBs of WT seeds showed typically spherical and ovoid structures and were distributed mostly between protein bodies at the periphery of the cells (Fig 6). In contrast, OE seed cells contained apparently smaller OBs than those of WT (Fig 6).

### Overexpression of *GmOLEO1* affected the expression of genes related and unrelated to oil synthesis

**Fig 7:**
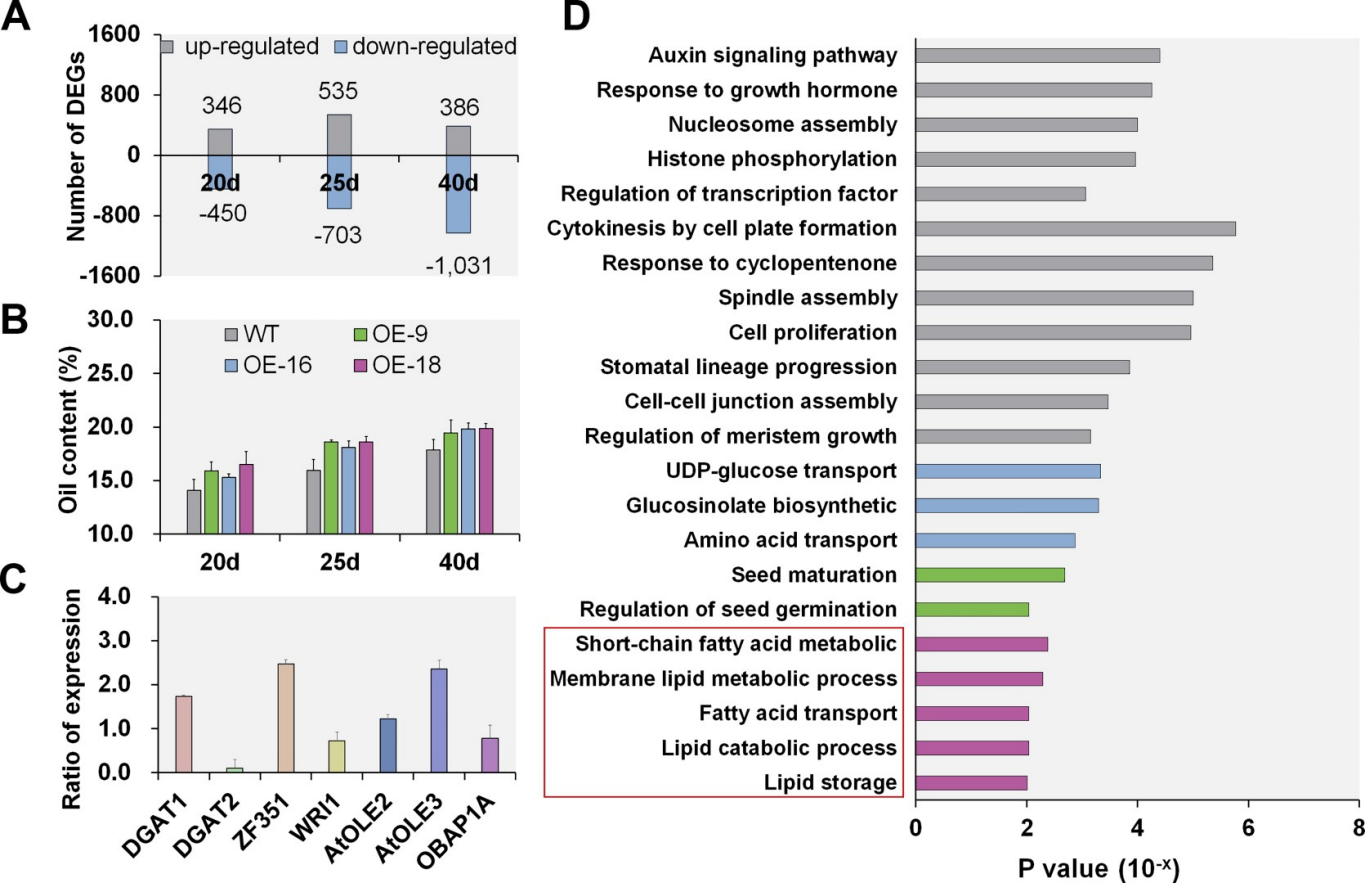
Comparative transcriptomic analysis of developing seeds between the OE lines and WT at different seed development stages. A, Histograms showing the differentially expressed genes (DEGs) in OE seeds relative to WT seeds at 20, 25 and 40 DAF, respectively. B, Comparison of oil content accumulation between the OE and WT seeds at 20, 25 and 40 DAF (*P* < 0.01). C, Expression analysis of the putative genes involved in plant oil biosynthesis in OE-9 seeds at 40 DAF. Error bars are SD. The Y-axis represents the ratio of expression of a gene in the OE lines relative to WT, D, GO enrichment analysis for the DEGs between the OE and WT seeds at 40 DAF. Purple, green, and blue columns represent the enriched GO terms associated with oil metabolism pathway, seed germination, and protein metabolism pathway, respectively.

To better understand the molecular mechanism by which *GmOLEO1* increased oil accumulation in soybean seeds, we compared the transcriptomes of OE and WT seeds at three seed development stages (20, 25 and 40 DAF) using RNA-seq analysis. In total, 796, 1238, and 1417 differentially expressed genes (DEGs) were identified by comparing OE with WT seeds at 20, 25, and 40 DAF, respectively (Fig 7A, S6–S8 Tables). The RNA-seq result was validated by *q*PCR analyses of 16 randomly selected genes (S9 Table, *R*^*2*^ = 0.84). We observed a trend toward increasing numbers of DEGs as DAF increased (Fig 7B). This increasing trend in the number of DEGs is consistent with the pattern of oil content increase in seeds as DAF increases (Fig 7B). This result indicated that overexpression of *GmOLEO1* resulted in significant changes in the transcriptomes in the developing OE seeds, and the changes became more dramatic as the seeds developed. To understand the biological processes in which *GmOLEO1* participates, we performed Gene Ontology (GO) enrichment analysis for these DEGs. In addition to the enrichment of GO terms associated with the regulatory pathways essential for plant growth and development, such as seed development and germination, amino acid and sucrose metabolism, and response to growth hormone, we found that GO terms associated with linoleic acid metabolism, fatty acid transport, lipid metabolism and storage were also significantly enriched for these DEGs (Fig 7D).

The RNA-seq results were further verified by the increased expression of several known genes participating in TAG biosynthesis in OE seeds as shown by *q*PCR, such as diacylglycerol acyltransferase (DGAT1) [18], wrinkled 1 (*WRI1*) [29], zinc-finger protein (*GmZF351*) [17], two *Arabidopsis OLEO* orthologs (*AtOLE2* and *AtOLE3*) [30], and oil body associated protein 1 (*OBAP1A*) [31] (Fig 7C), indicating that the expression of these genes may be affected by *GmOLEO1* overexpression. These results indicated that overexpression of *GmOLEO1* promoted the expression of TAG biosynthesis-related genes and led to the enhancement of TAG biosynthesis.

### *GmOLEO1* underwent artificial selection for increased seed oil accumulation

**Fig 8:**
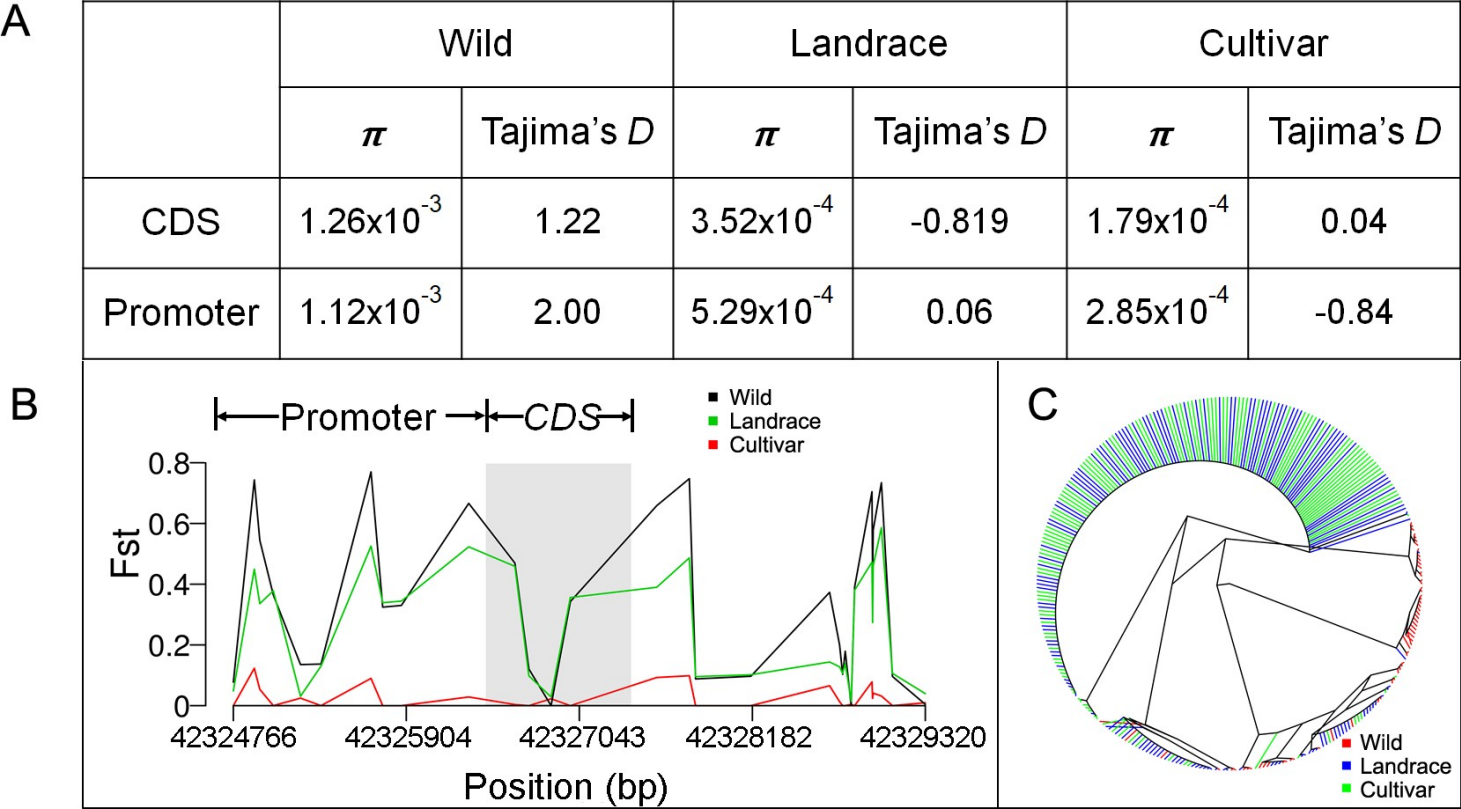
Diversity and evolutionary analyses of *GmOLEO1* between wild, landrace and cultivated soybeans. A, Gene diversity (π) and Tajima's D of *GmOLEO1* in the three groups. B, *Fst* in the promoter and coding sequence (CDS) of *GmOLEO1* between the three groups. C, A phylogenetic tree comprising the three groups constructed using the SNPs in the promoter and CDS region of *GmOLEO1*.

Because higher expression of *GmOLEO1* in cultivated soybean than in wild soybean was observed, we hypothesized that the variations in its promoter region were under selection. Statistical analyses were performed using a large population of 302 soybean accessions [24]. We first evaluated *Fst* for different comparisons, including wild soybean vs. cultivar, wild soybean vs. landrace, and cultivar vs. landrace. The results showed that the *Fst* between wild vs. cultivated soybean is considerably higher than that between cultivar vs. landrace, especially in the promoter region (Fig 8A and 8B). The nucleotide diversity (π) analysis showed that π was higher in wild soybean than cultivated soybean in the promoter region and the coding region (Fig 8A). Tajima's *D* in the promoter region was 2.00, 0.06 and -0.84 for wild, landrace and cultivar, respectively, while Tajima's *D* in the coding region was 1.22, -0.819, and 0.04 for wild, landrace and cultivar, respectively (Fig 8A), implying that positive selection had occurred in the promoter region. A phylogenetic analysis using variants in the promoter and coding regions identified three clusters, corresponding to wild soybean, landrace, and cultivar (Fig 8C). Taken together, these results indicated that the promoter region was subjected to artificial selection during domestication.

## Discussion

It is known that seed oil content has been subjected to artificial selection targeting higher oil content [25]. This finding was further validated by our study, in which a significant difference in seed oil content between cultivated and wild soybeans was observed (Fig 1D). Unlike other domestication traits in soybean, such as stem growth habit [32] and pod shattering [33], soybean oil content is highly complex; it is regulated by many genes of small effect and is easily influenced by various environmental factors [1]. Our GWAS study across multiple environments allowed us to identify a new environmentally stable QTL, *GqOil20*, and an underlying candidate gene, *GmOLEO1* that is capable of increasing seed oil content in soybean. Notably, the *GmOLEO1* locus was previously identified as a possible candidate for an eQTL associated with seed oil accumulation [34], and it is physically close to other oil-related QTLs previously identified by linkage mapping [21–23]. *GmOLEO1* may have been identified in this study because the corresponding alleles were fixed with respect to oil variation during domestication. Given the complexity of oil metabolism, the observed phenotypic variation (23.7%) could be due to the combined effects of *GmOLEO1* and other genes at this locus. Other oleosin genes, regardless of sequence variation, with increased expression at the gene or protein level [35–36] during seed filling/maturation may also substantially affect oil accumulation. Nevertheless, our study further functionally verified that human-selected *GmOLEO1* might be involved in seed oil accumulation, possibly by indirectly affecting oil biosynthesis via efficient feedback.

Our study and previous studies have indicated that the improvement of seed oil content in soybean during domestication was achieved by artificial selection of multiple major genes, and some of those genes may not be directly involved in oil biosynthesis, such as *B1* [37] and *GmZF351* [17]. In contrast to previous studies that identified oil-related genes using a reverse genetic approach, *GmOLEO1* was pinpointed in an artificially selected locus, *GqOil20*, using an integrated strategy of high-density genetic mapping and genomics. Haplotype and expression analyses of *GmOLEO1* between cultivated and wild soybean in our study provided additional evidence of selection at the *GmOLEO1* locus. This artificially imposed selection pressure on the expression of *GmOLEO1* could be an important factor affecting the observed difference in oil accumulation in soybean, because the overexpression of Hap2 of *GmOLEO1* resulted in enhanced oil accumulation in transgenic soybean (Figs 1G and 4F). Whether other genes in this block have functions associated with oil accumulation requires further determination.

Seed yield and quality represent two of the most important traits in soybean improvement. Breeding soybeans with high oil stability across environments while maintaining protein content and yield has been difficult due to the complex genetic architecture of oil regulation. In our study, *GmOLEO1* was functionally identified as a candidate for the environmentally stable QTL *GqOil20*. Overexpression of *GmOLEO1* significantly elevated oil content and the percentage of polyunsaturated FAs without detriment to the overall plant performance, especially yield, in our preliminary study, making *GmOLEO1* a promising candidate gene for use in breeding high-oil soybeans with improved levels of healthy polyunsaturated FAs. Although Hap2 (Williams 82-type) is not the most favored haplotype for increasing oil accumulation, enhanced seed oil accumulation was observed in this study, indicating that the *GmOLEO1* allele in Hap2 enhanced oil accumulation independent of the amino acid substitution (Ala265Pro). One possible reason for this finding is that the substitution, which does not change hydropathy, may not affect the secondary structure of oleosins in OBs. The strong correlation between Hap1 and high expression levels of *GmOLEO1* alleles (Fig 1) suggests the importance of unique variation (Hap1) in the promoter region in enhancing the expression of *GmOLEO1*. The discovery of the molecular function DNA marker, Indel P237167, from the unique variation in the promoter of *GmOLEO1* will facilitate marker-assisted selection (MAS) in soybean high-oil breeding programs.

The importance of oleosins in lipid accumulation and oil body formation in seed plants has been gradually recognized over the past three decades, and it has demonstrated an important role in the maintenance of OBs and preventing them from coalescence [9]. The *Arabidopsis* genome contains 17 oleosin genes [38], of which *AtOLE1* has been reported to be involved in lipid biosynthesis [39]. In soybean, 13 putative oleosin genes were found in the *G*. *max* reference genome, while only *GmOLEO1* colocalized with the associated SNP (AX-93661332) in *GqOil20* in our study (S10 Table). The overexpression of *GmOLEO1* showed consistent results, as observed in *AtOLE1*, revealing conserved functions between *GmOLEO1* and *AtOLE1* in increasing oil accumulation. Despite being rarely studied in other species, the high similarity in amino acid sequence and structural domains (Fig 2A and 2B) suggests that GmOLEO1-like proteins from the Faboideae, Brassicaceae, and grass clades might have a conserved function in determining OB size but lineage-specific roles [7]. For example, expression of *GmOLEO1* correlated with oil content in our study, and OE seeds with increased oil content contained smaller OBs; conversely, the expression of oleosin genes was independent of oil content in maize, and a high-oil maize strain contained larger, more spherical OBs than did low-oil maize [40]. The conserved role of *OLEO*s from various plant species in enhancing oil accumulation suggests that *GmOLEO1* orthologs have considerable potential for oil improvement in other oil-producing crops.

It has been demonstrated that oleosins have important functions in OB formation, stabilization, and transgenic addition of oleosin increased oil content in *Arabidopsis*, *Brassica* and yeast [10, 31, 41–42], but oleosins’ role in increasing oil accumulation in soybean seeds has rarely been reported previously. In addition to these potentially cross-species functions in determining the size of OBs and affecting oil accumulation, our study showed that increased oleosins resulted in apparent reductions in OB size and increases in OB number in seed cells (Fig 6) and increased seed oil contents (Fig 2), in agreement with a study in which suppression of OLEO1 resulted in larger OBs and reduced total lipid levels in seeds [9–10]. Oil accumulation was gradually regulated as the seeds matured, possibly due to a gradual increase in the expression of *GmOLEO*1, which was concomitant with the enhanced TAG metabolism during seed maturation identified by RNA-Seq (Fig 7). Thus, it is logical that a positive correlation between oil content and *GmOLEO1* expression was observed in our study (Fig 1G). A similar correlation has also been observed in *Brassica napus*, where the expression levels of *OLEO*/oleosin in high-oil genotypes were considerably higher than those in low-oil seeds [41]. The higher expression levels of *GmOLEO1* in high-oil soybean varieties might be attributed to the unique variation present in the promoter of Hap1. The presence of putative seed-maturation-related cis-elements (abscisic acid (ABA) response and seed regulation, Fig 1F) in the promoter region of *GmOLEO1* may be responsible for its exclusive expression during seed maturation.

In addition to stabilizing the structures of lipid droplets (LDs), oleosins also serve other functions, including enzymatic and signaling roles. Some of these proteins are ubiquitous in cells with and without LDs, thus exerting broader functions in seeds and other organs [43]. In peanut, oleosin3 (OLE3) was shown to exhibit bifunctional activities and was phosphorylated by STYK (AhSTYK) to regulate MGAT and PLA2 activity; it could be involved in the biosynthesis and mobilization of TAGs during seed maturation and germination [44]. However, a recent report showed that the bifunctional enzymic motifs are present in only peanut oleosins and not in those of other plants [7]; thus, another possibility is that oil accumulation increases as a result of *GmOLEO1* overexpression, which might lead to efficient feedback by producing smaller OBs [45]. The detailed mechanisms underlying the regulation of gene expression by GmOLE1 must be deciphered in future work.

The level of oleosin itself is regulated during seed development and germination. When seeds germinate, oleosin degradation occurs prior to OB degradation. A recent study revealed that the ubiquitin binding protein PUX10 and division cycle 48 homolog A (CDC48A) are core components of an LD-associated ERAD-like degradation machinery, which facilitates the dislocation of oleosins from LDs [46–47]. In our study, faster seed germination of OE lines might be associated with higher levels of some oleosin-degradation proteins (e.g., PUX10 and CDC48A) [46–47], but this hypothesis remains to be experimentally determined.

Based on the results of our preliminary study and a previous finding [9], we proposed that the biosynthesis of TAGs was enhanced in the OE lines, possibly because of the affected TAG metabolic pathway, as a result of increased expression of *GmOLEO1*/oleosins. Smaller OBs gradually accumulated as the newly produced TAGs reached the minimum size that could be completely covered by the increased number of oleosin proteins, given that oleosins serve as surfactant to prevent OBs from coalescence [10, 48]. Thus, increased oleosin production in OE seeds, which resulted in reduced size but increased turnover of OBs during seed maturation, could be a more efficient way to use the limited intracellular space than larger OBs, leading to increased total oil content in OE seeds.

## Material and methods

### Plant materials and field experiments

The association panel for GWAS consisted of a diverse collection of 219 soybean accessions (including 195 landraces and 24 elite varieties) originating from 26 provinces across six different agroecological regions in China, ranging from latitudes 53 to 24°N and longitudes 134 to 97°E [49]. Field experiments were performed in the 2009, 2011, 2012, 2013 and 2014 growing seasons at four different geographic locations as previously described [50]. Briefly, soybean plants were examined under field conditions at the following experimental stations: Jiangpu Experimental Station of Nanjing Agricultural University (32.1°N 118.4°E), Nanjing, in 2009 (designated as Environment 1, E1); Maozhuang Experimental Station (34.8°N 113.6°E) of Henan Agricultural University Zhengzhou, in 2009 (E2) and 2011 (E3); the Fangcheng Experimental Farm (33.2°N 112.9°E) of Henan Agricultural University in 2012 (E4), and Yuanyang Experimental Station of Henan Academy of Agricultural Sciences, Zhengzhou, in 2013 (E5) and in 2014 (E6). A randomized block design was used for all field trials. In all environments, each accession was planted in a three-row plot, with each row 200 cm long and 50-cm row spacing.

### Phenotyping and genotyping

Mature soybean seeds were harvested and air-dried, and fully filled seeds were used for oil content measurement. Measurement of soybean oil, protein, and FA components was conducted using a near infrared spectrophotometer (NIR) seed analyzer (DA7200, Perten Instruments, Huddinge, Sweden) as previously described [51]. This association panel was genotyped using the NJAU 355K SoySNP array as previously described [26], and a total of 292,035 high-quality SNPs were used for association mapping.

### Phenotyping analysis

Phenotypic data for soybean seed oil across different environments were subjected to an ANOVA using the PROC GLM (general linear model) mixed model of SAS version 9.2 (SAS Institute, 2002). The linear statistical model includes the effects of genotype, environment and the environment × genotype interaction. The BLUP for each line was calculated with PROC MIXED in SAS (SAS Institute, 2002) and used as the phenotypic input for the subsequent GWAS. The violin plot was drawn using the R package vioplot [52]. The heritability of oil content was calculated using *h*^*2*^ *= Vg/ (Vg+Ve)*, where *Vg* and *Ve* represent genetic and environmental variation, and each term was extracted from the ANOVA results.

### Genome-wide association study

GWAS was conducted using the compressed mixed linear model with TASSEL 5.0 [53, 54] using SNP with minor allele frequency greater than 0.05, and the threshold was determined with Bonferroni threshold of ≤ 4.95 × 10^−6^ (*P* = 1/n) [55], where n is the SNP number used in GWAS. The population structure and the relatedness were described previously [26]. The Manhattan plot was drown using the R package qqman [56]. The LD heat map was plotted using the LDheatmap R package [57].

### Quantitative real-time PCR

Expression of the candidate gene was examined in different soybean tissues, including roots, shoots, leaves, flowers, pods, and developing seeds at different developmental stages (10, 20, 25, 30 and 40 days after flowering). Total RNA was isolated from the tissues using the RNAsimple Total RNA Kit (TaKaRa, Japan), and 1 μg of RNA was treated with 10 units of RNase-free DNase I (TaKaRa) prior to cDNA synthesis. The first strand of cDNA was synthesized using the SuperScript III First-Strand Synthesis System (Invitrogen, USA) following the manufacturer's instructions. Gene expression was determined using the Bio-Rad CFX96 Touch Real-Time PCR System (Bio-Rad, California, USA). The PCRs contained 5 μL of the first-strand cDNA, 0.5 μL of 10 μmol L^−1^ gene-specific primers (S11 Table), and 10 μL of Real-Time PCR SYBR Mix (PC3302; Aidlab). The PCR conditions were as follows: 94°C for 3 min and 40 cycles at 94°C for 15 s and 60°C for 15 s. The soybean *tubulin* gene (GenBank: AY907703.1) was amplified as an internal reference, and a negative control reaction was performed using water instead of cDNA. Three biological replicates per sample were used, and each reaction was performed in triplicate.

### Dual luciferase assay

In the protoplast transient expression experiments, the dual luciferase assay vector pGreenII 0800-LUC was used to analyze the activity of the different promoters. This vector contains a firefly luciferase (LUC) reporter gene that can be driven by the target promoter and a Renilla luciferase (REN) reporter gene driven by 35S. The purified DNA fragment of the target promoter was fused with the LUC reporter gene in the vector digested with HindIII and SalI enzymes to construct the recombinant vector. The vector pGreenII 0800-LUC without promoter insertion before the LUC reporter gene was used as a control. The recombinant vector and the control were individually transformed into Arabidopsis protoplasts via PEG-calcium transfection. The isolation of Arabidopsis protoplasts and protoplast culture were performed according to standard protocols [58]. The ratio of LUC and REN activity (LUC/REN) was used to reflect the activity of the target promoter. The LUC/REN value was determined using the dual luciferase reporter assay system (Promega, USA).

### Vector construction and generation of transgenic plants

The complete coding sequence of *GmOLEO1* was amplified from the cDNA of Williams 82 by regular PCR using gene-specific primers (S11 Table). The PCR product was subcloned into the pMD-19 T vector (TaKaRa, Japan) for sequence verification. The verified *GmOLEO1* sequence was then cloned into the dicotyledon expression vector pCAMBIA3300, which contains a selection marker gene, phosphinothricin acetyltransferase (*bar*), using the ClonExpress Entry One Step Cloning Kit. The resulting recombinant pCAMBIA3300-*GmOLEO1* construct was transformed into Williams 82 via the *Agrobacterium tumefaciens*-mediated soybean cotyledon node transformation system as previously described [59]. Extraction of genomic DNA from the leaves of PPT-resistant plants and nontransformed plants was performed using the cetyltrimethylammonium bromide (CTAB) method [60]. Transformants were verified by leaf-painting assay with herbicide phosphinothricin (PPT), PCR analysis for the presence of introduced *GmOLEO1* and *bar* (482 bp), and LibertyLink strip detection for the expression of the *bar* gene using the QuickStix Kit (EnviroLogix Inc., ME, USA) were considered positive transgenics for further analysis. For LibertyLink strip detection, a total of 100 mg leaf tissue was collected and ground completely in the bottom of a conically tapered 1.5 ml tube by pestle rotation, followed by adding 0.5 mL of extraction buffer and a strip into the tube. After ten minutes, strips containing only the control line were negative for PAT protein expression, while those with two lines (control line and test line) were positive for PAT protein expression [61].

### Subcellular localization and colocalization assay

The full-length *GmOLEO1* cDNA was amplified and cloned into the pBWA (V)HS-osgfp vector to obtain the pBWA(V)HS-osgfp-35S::*GmOLEO1*-*GFP* construct under control of the cauliflower mosaic virus (CaMV) 35S promoter (Biorun Co., Ltd). The binary vector 35S::*GmOLEO1*-*GFP* was transiently coexpressed in the leaves of *Nicotiana benthamiana* via *agro-*infiltration. Then, the tobacco leaf epidermal cells *agro*-infiltrated with the *GmOLEO1*-GFP construct were stained with Nile Red, a lipophilic dye used to visualize OBs [4]. Fresh leaves were placed in a solution containing Nile Red stock (100 mg/mL dimethyl sulfoxide) diluted 100× with 1×PBS for 10 min and washed with PBS twice for 30 s each time. Fluorescence signals were detected using a confocal laser scanning microscope (Nikon C2-ER, Japan) 2–3 days after infiltration. GFP, mKate and Nile Red were excited at 488, 561 and 559 nm, and their emission was detected at 510 to 540, 580 to 620 and 570 to 670 nm, respectively. All of these fluorescence experiments were independently repeated at least three times.

### Western blot analysis for *GmOLEO1*

Immunogenicity peptides of *GmOLEO1* protein were predicted by bioinformatics analysis. The sequences of the peptides were as follows: MAELHYQQQHQYPHR and KDYGQQQISGVQAS. The peptides were commercially synthesized and purified (Wuhan GeneCreate Biological Engineering Co., Ltd, China). Two male Japanese White rabbits were used for the immune procedure. Next, a polyclonal antibody of GmOLEO1 protein was separated and purified for immunoblot analysis. Proteins of fresh soybean seed were extracted by Triton X-100 lysate (0.5%). Then, 30 μg of protein extracts mingling with 2× SDS-PAGE sample loading buffer (Solarbio, Beijing, China) were loaded and subjected to SDS-PAGE. Afterward, protein bands were transferred onto polyvinylidene fluoride (PVF) membranes (Solarbio, Beijing, China). The membranes were blocked with 5% skimmed milk powder solution for 2 h at room temperature, followed by incubation with a polyclonal antibody against GmOLEO1 diluted to 1:10000 in phosphate-buffered saline overnight at 4°C. Finally, the blot was detected with horseradish peroxidase (HRP)-conjugated goat-anti-rabbit secondary antibody (Santa Cruz Biotechnology, USA) for another 1 h. The protein bands were visualized using a chemiluminescence system (Pierce, Rockford, Illinois, USA).

### RNA-seq analysis of transgenic soybean

Transcriptomes were compared between pooled OE seeds at 20, 25, and 40 DAF, respectively, with the WT seeds at the corresponding developmental stage. For each time point, two developing seeds from each of the three OE lines (OE-9, -16 and -18) were collected and pooled as one biological replicate, and three biological replicates were used per sample. Library construction was performed as previously described [62]. The library was sequenced with the Illumina HiSeq 2500 analyzer at Biomarker Technologies (Beijing, China), producing 200-bp paired-end reads. An average of 6.47 gigabases of clean data per sample was generated. Differential gene expression was determined using the DESeq R package [52]. A gene with an adjusted *P* < 0.05 and a fold change (FC) >1.5 were defined as DEGs. Enrichment analysis of Gene Ontology of biological pathways (GOBPs) was performed using the GOseq R packages [63] to compute *P* values that indicate the significance of each GOBP being represented by the genes. GOBPs with *P* < 0.01 were identified as enriched biological processes.

### Microscopy analysis

Fresh immature soybean seeds harvested at 25 and 40 DAF were fixed in FAA fixation solution for at least 24 h. The main experimental steps for Oil Red O staining are as follows: cutting the whole sample into small blocks, removing excess water with tissue paper, immersing the small tissue blocks in Oil Red O (Servicebio, G1016, Wuhan, China) solution and incubating at 37°C for 60 min. Excess staining solution was removed by rinsing with tap water. The stained tissue blocks were immersed in 75% ethanol for 30 min or until no fading occurred; then, they were preserved in 4% paraformaldehyde and kept in the dark. Photos were taken using a digital camera (Canon 7D). The fixed tissue samples were embedded with OCT compound (Sakura, Japan). Frozen sections (8–10 μm) were obtained with Cryostat Microtome (Thermo, CRYOSTAR NX50, USA) and mounted on a prechilled glass slide. The frozen sections were stained with 0.1% Nile Red (Servicebio, G1073, Wuhan, China) and Oil Red O (Servicebio, G1016, Wuhan, China). Image observation for Nile red staining was performed using a Nikon confocal scanning microscope (Nikon, C2, Japan). The excitation wavelength was 488 nm, the emission wavelengths were 593–654 nm, and the OBs were imaged at 800× magnification. Oil Red O staining was imaged using an optical microscope (Nikon, Eclipse Ci, Japan) at 800× magnification.

Tissues (1×3 mm^3^ in size) of developing soybean seeds were fixed in 2.5% glutaraldehyde buffered with 0.1 M phosphate buffer (pH 7.2) for 12 h. Postfixation was subsequently conducted in 1% osmic acid in 0.1 M phosphate buffer (pH 7.2) for 5 h. The blocks were then washed, dehydrated through an ethanol series of 30–100%, and embedded in EMbed 812 media. The samples were cut into 1 μm slices using an ultramicrotome (Leica UC7, Germany), stained with alkaline toluidine blue O solution (Servicebio, G1032, Wuhan, China), and then imaged (800×) using an optical microscope (Nikon, Eclipse Ci, Japan). For transmission electron microscopy (TEM), the samples were cut into 60 nm slices using an ultramicrotome (Leica UC7, Germany) and then separately stained with uranyl acetate and lead citrate for 15 min. The slice samples were photographed under a TEM (HT7700, Hitachi, Japan).

### Gene resequencing and haplotype analysis of *GmOLEO1*

The 2.3-kb genomic region spanning from 1,500 bp upstream from the translation start codon (ATG) to the 3’-untranslated region (UTR) of *GmOLEO1* was sequenced and analyzed. Haplotype analysis was performed by resequencing this region in 20 high-oil, 20 low-oil and 10 moderate-oil accessions. All primers (S11 Table) used in this study were designed using the Primer 3 online tool (http://frodo.wi.mit.edu/primer3/). All sequences were verified manually, and all observed polymorphisms were reverified by resequencing of another amplicon. All the verified sequences were aligned using ClustalX version 1.83 [64]. The polymorphism data were analyzed using DnaSP version 4.10 [65] to identify sequence variation. Prediction of cis-elements in the promoter region was carried out using the online web tool PlantCARE [66].

### Fatty acid component analysis

FA components in soybean seeds were analyzed as previously described [17]. Briefly, 10 mg fine powder of soybean seeds was used for FA isolation. FAs were extracted with 1 mL of extraction buffer (2.5% [v/v] H_2_SO_4_ in CH_3_OH) at 85°C for 1 h. The supernatant (500 μL) was mixed with 300 μL of hexane and 600 μL of 0.9% (w/v) NaCl. FA methyl esters were redissolved in 200 μL of ethyl acetate and analyzed immediately with a gas chromatography system (GC-2014; Shimadzu, Beijing, China). Peaks corresponding to each FA species were identified by comparison to a FA methyl ester analytical standard (Supelco, Poole, UK). Concentrations of FA species were normalized against the internal control heptadecanoic acid (Sigma-Aldrich, USA). Five biological replicates per line were analyzed in this experiment.

### Germination test

The seeds were surface-sterilized with chlorine gas for 4 h prior to germination in darkness in Petri dishes (90 mm in diameter) on two sheets of filter paper moistened with deionized water (15 seeds per Petri dish). Germination tests were carried out in an incubator (MGC-400B, YIHENG, Shanghai, China) equipped at 25°C with 75% humidity. The filter paper was replaced once a day, and germinated seeds with healthy roots were counted. Root length was measured using a ruler at 2, 3 and 7 days postgermination. Three replicates per treatment were performed.

### Gene diversity analysis

The published whole genome sequencing data were used for gene diversity analysis [25]. VCFtools was used to estimate gene diversity (*Fst*, nucleotide diversity (π) and Tajima’s *D*) [67]. SNPRelate combined with APE was used to construct the phylogenetic tree [68, 69].

## Supporting information

S1 Fig Oil variation and GWAS results. **A,** Oil content variation in all 219 accessions across six environments. **B,** Quantile-quantile plot of the GWAS results under a general linear model (GLM) and mixed linear model (MLM, Q+K). **C,** Phenotypic distribution of the oil trait across six environments. E1-E6 denote the oil content in the corresponding environments.
(TIF)

S2 Fig Manhattan plots showing the GWAS results of soybean oil content across six environments (MLM, Q+K, *P* < 4.95 × 10−6). The x-axis shows the 20 soybean chromosomes, and the y-axis shows the significance expressed as a −log_10_*P* value. The quantile-quantile plot corresponding to the GWAS (MLM, Q+K, *P* < 4.95 × 10^−6^) result in each environment is given beside the Manhattan plot.
(TIF)

S3 Fig A regional view of *F_ST_* between the *G. max-G. soja* group and *G. max* only at the three significant association loci. The dotted line represents the 95% tails for the empirical distribution of *F*_*ST*_ statistics.
(TIF)

S4 Fig Identification of positive transgenic plants. (A-C) Identification of positive transgenic plants by leaf-painting assay (A), polymerase chain reaction (PCR) verification (B) and strip detection for the presence of the selective *bar* gene (C).
(TIF)

S5 Fig Hierarchical clustering of differentially expressed genes in the transgenic lines relative to WT plants. The indicated scale is the log_2_ value of the normalized level of gene expression.
(TIFF)

S1 Table Phenotypic variation analysis for oil content of the 219 accessions in six different environments. (XLSX)

S2 Table GWAS result for oil content in soybean seeds across six different environments and their BLUP. (XLSX)

S3 Table Candidate genes within the 50-kb region located on either side of the peak SNP AX-93910018. (XLSX)

S4 Table Comparison of oil, protein, and yield-related traits between OE lines and WT. (XLSX)

S5 Table Fatty acid composition analysis between OE and WT seeds. (XLSX)

S6 Table Differentially expressed genes identified in developing seeds between the OE lines and WT at 20 DAF. (XLSX)

S7 Table Differentially expressed genes identified in developing seeds between the OE lines and WT at 25 DAF. (XLSX)

S8 Table Differentially expressed genes identified in developing seeds between the OE lines and WT at 40 DAF. (XLSX)

S9 Table Relative gene expression determined by qPCR at 25 DAF and the list of primers used. (XLSX)

S10 Table Physical locations of the 13 *OLEO* genes in the soybean genome. (XLSX)

S11 Table Primers used in this study. (XLSX)
