# Supplementary figures and images for: Artificial selection on GmOLEO1 contributes to the increase in seed oil during soybean domestication
Source: PLoS Genet. 2019 Jul 10;15(7):e1008267. doi: 10.1371/journal.pgen.1008267 (PMC6645561; doi:10.1371/journal.pgen.1008267)

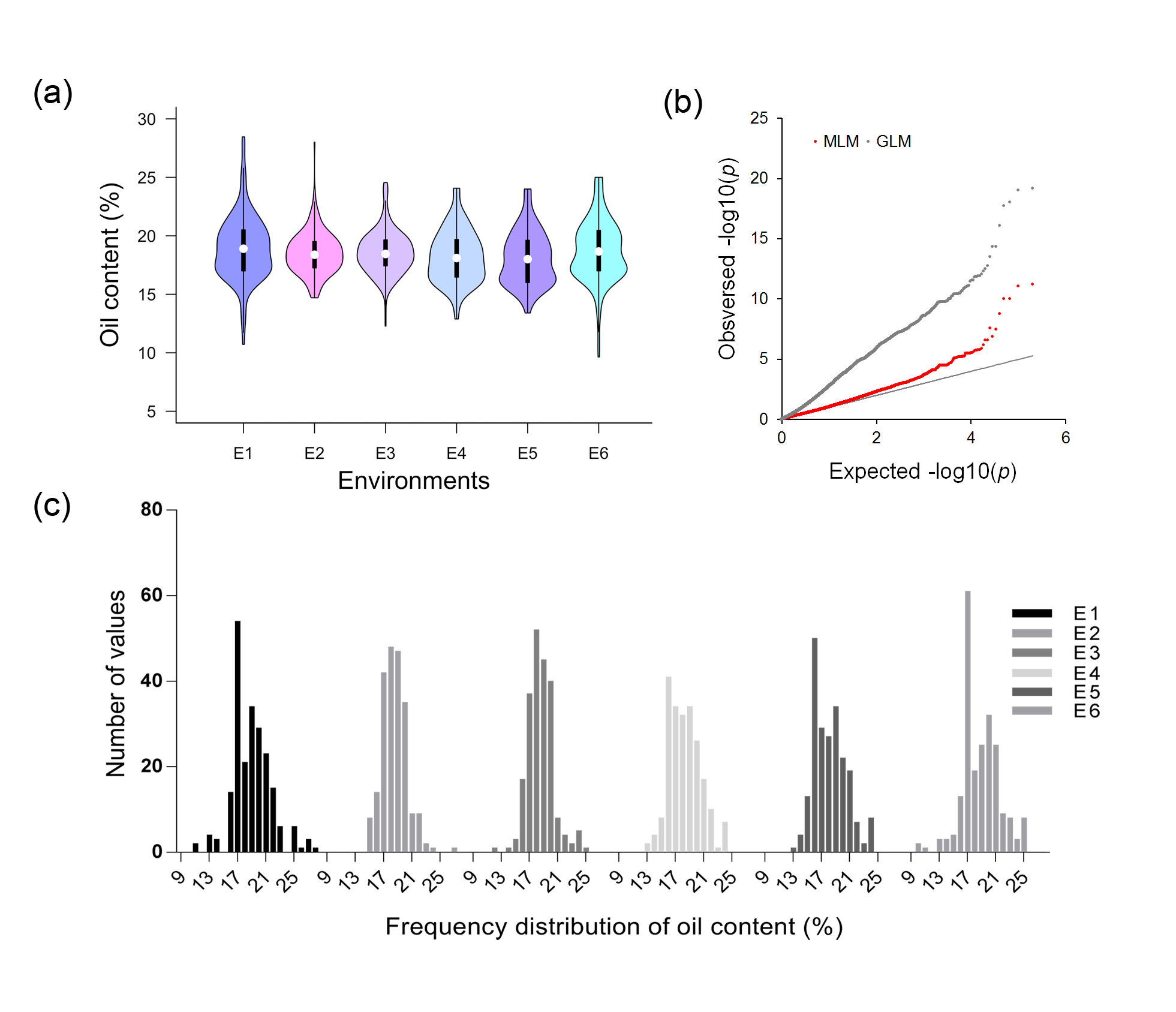

Supplement: S1 Fig — A, Oil content variation in all 219 accessions across six environments. B, Quantile-quantile plot of the GWAS results under a general linear model (GLM) and mixed linear model (MLM, Q+K). C, Phenotypic distribution of the oil trait across six environments. E1-E6 denote the oil content in the corresponding environments. (TIF) [file pgen.1008267.s001.tif]

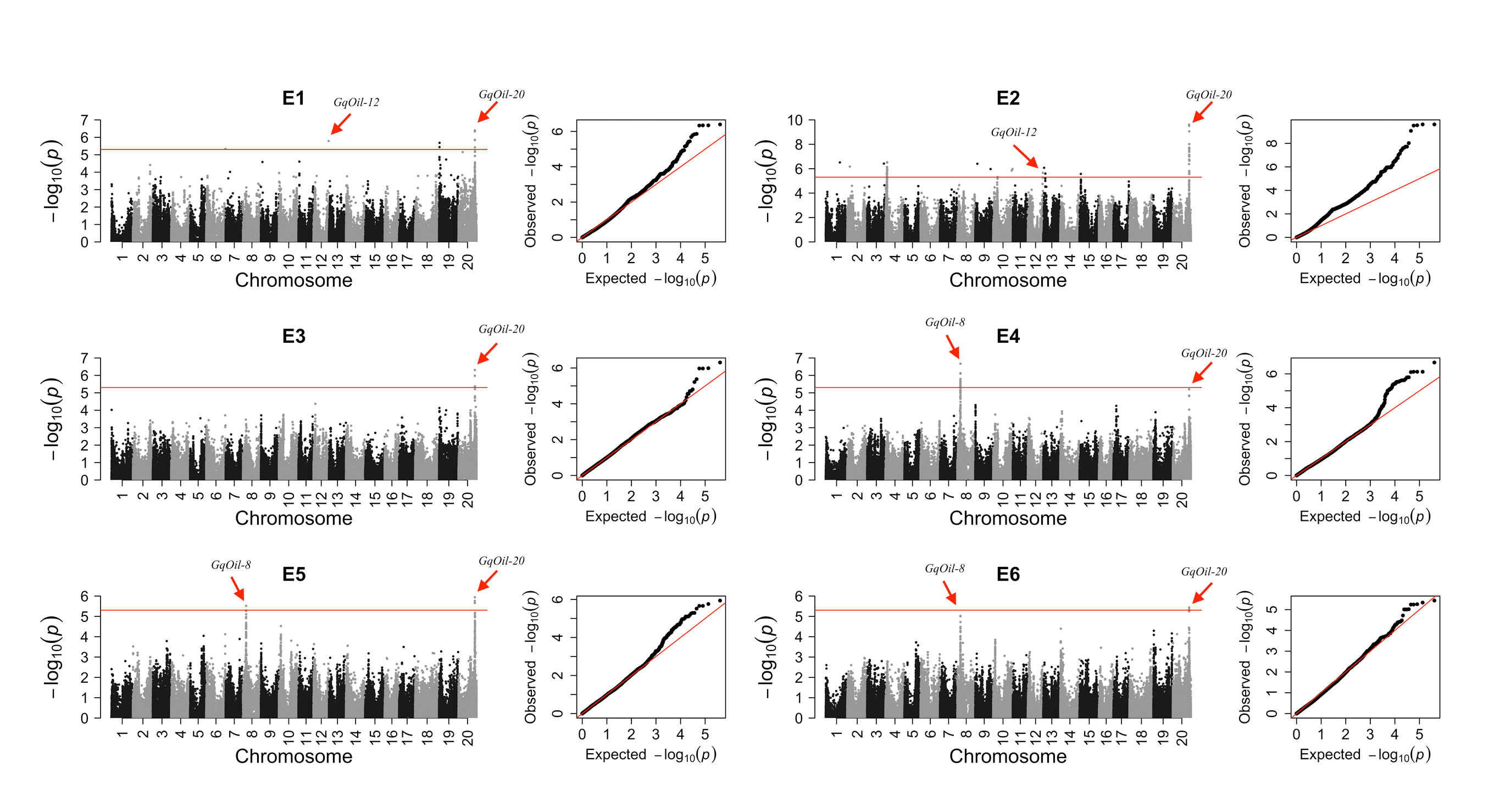

Supplement: S2 Fig — The x-axis shows the 20 soybean chromosomes, and the y-axis shows the significance expressed as a −log10P value. The quantile-quantile plot corresponding to the GWAS (MLM, Q+K, P < 4.95 × 10^−6^) result in each environment is given beside the Manhattan plot. (TIF) [file pgen.1008267.s002.tif]

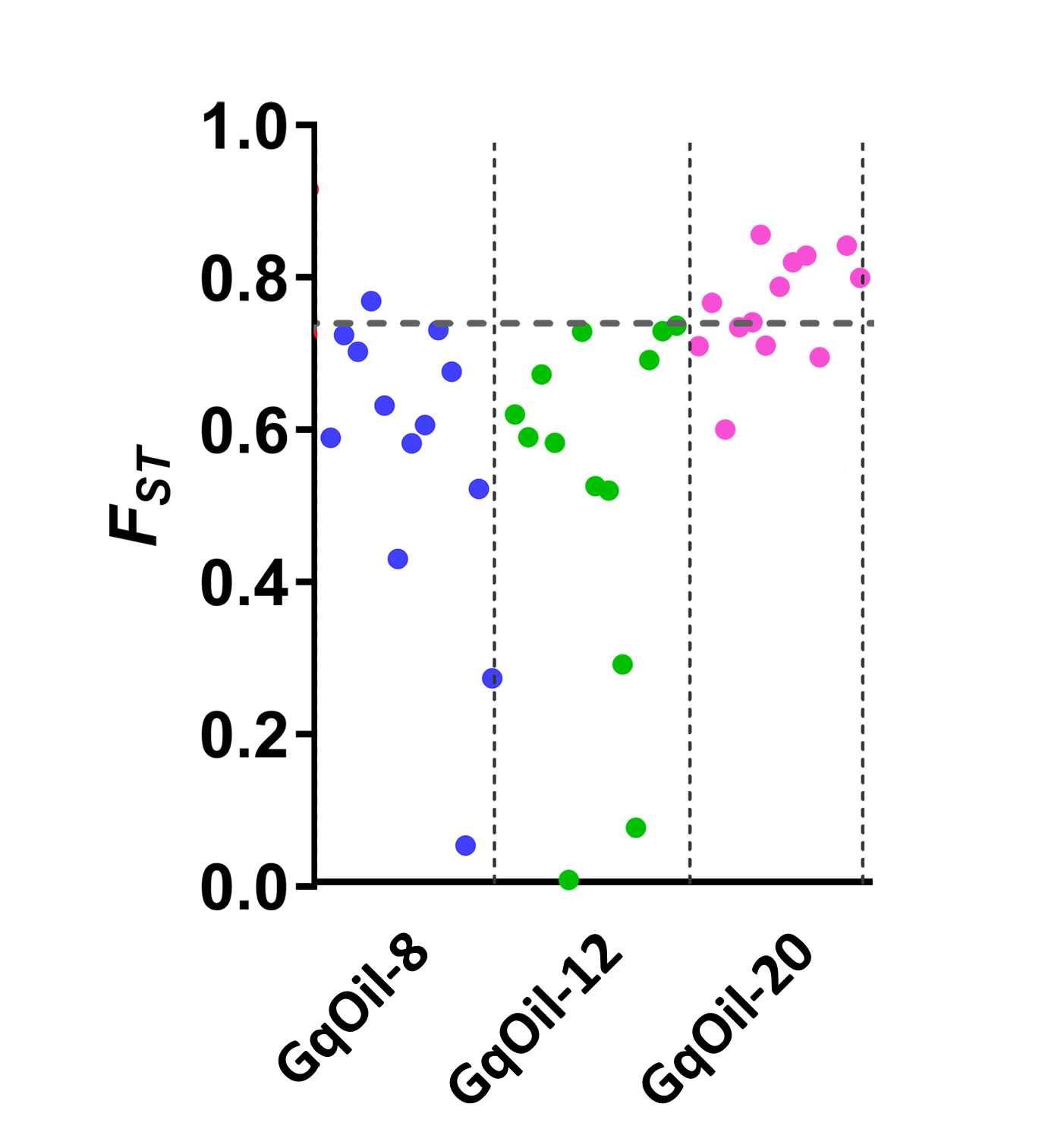

Supplement: S3 Fig — The dotted line represents the 95% tails for the empirical distribution of FST statistics. (TIF) [file pgen.1008267.s003.tif]

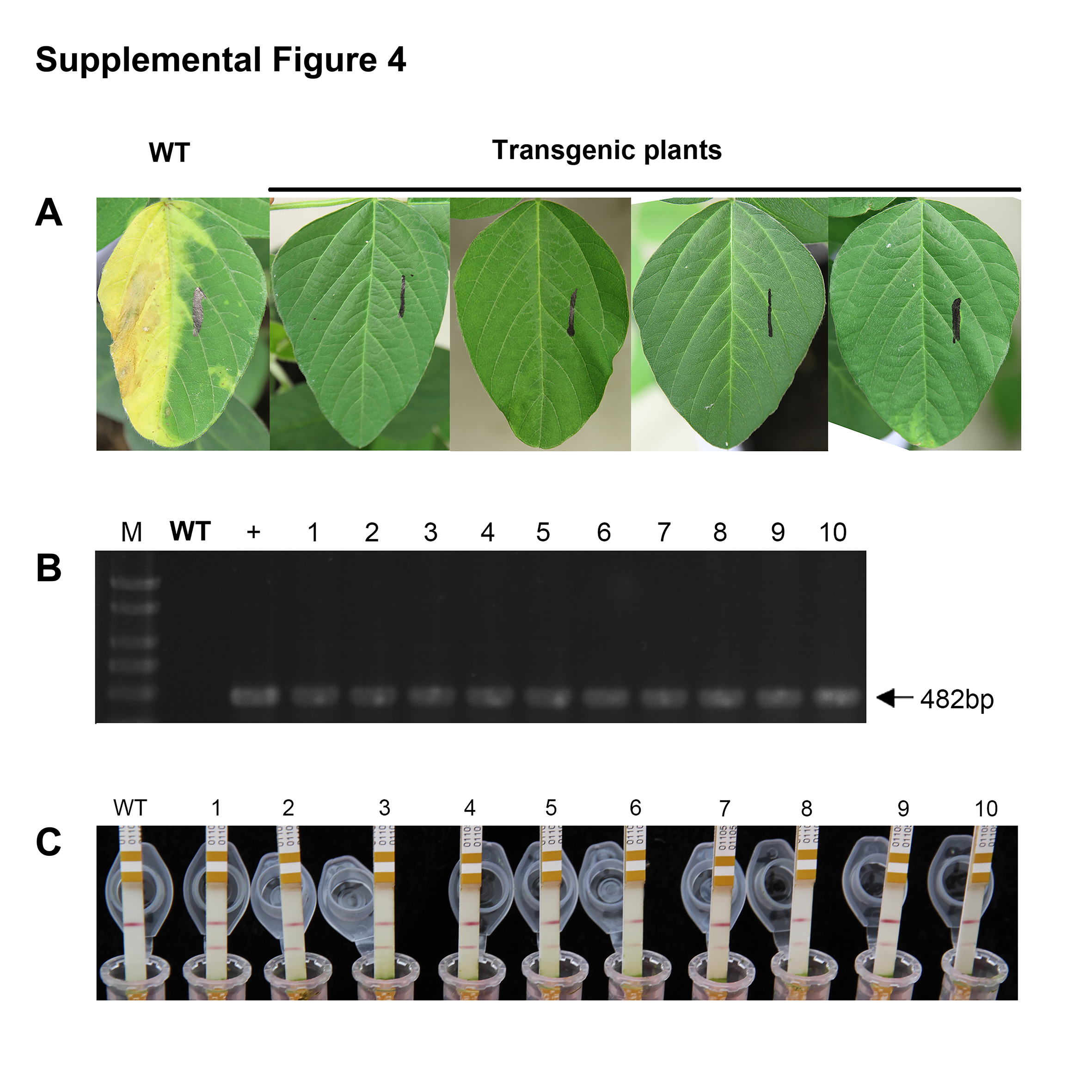

Supplement: S4 Fig — (A-C) Identification of positive transgenic plants by leaf-painting assay (A), polymerase chain reaction (PCR) verification (B) and strip detection for the presence of the selective bar gene (C). (TIF) [file pgen.1008267.s004.tif]

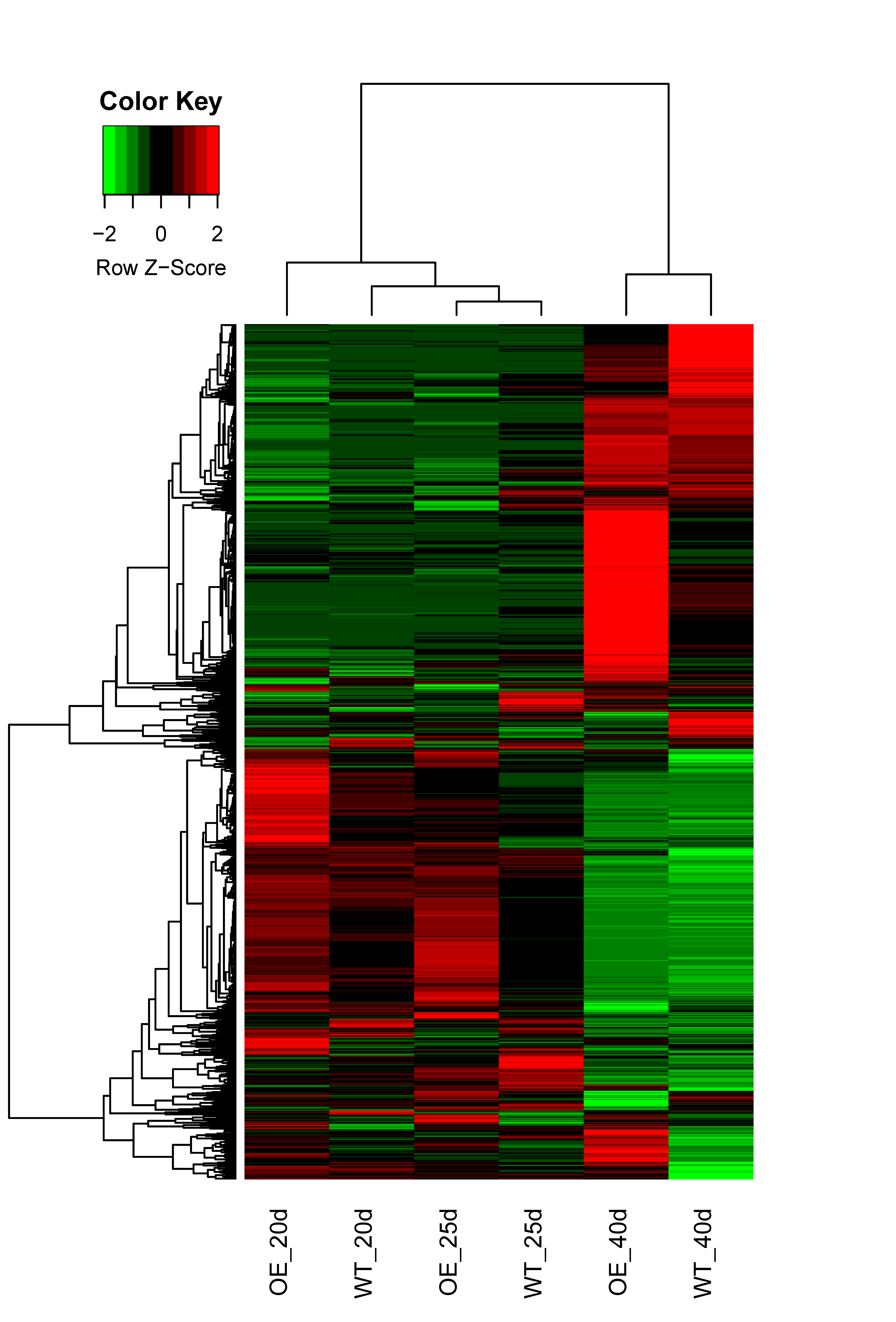

Supplement: S5 Fig — The indicated scale is the log2 value of the normalized level of gene expression. (TIFF) [file pgen.1008267.s005.tiff]
